# Evidence for a biphasic mode of respiratory syncytial virus transmission in permissive HEp2 cell monolayers

**DOI:** 10.1186/s12985-016-0467-9

**Published:** 2016-01-20

**Authors:** Tra Nguyen Huong, Laxmi Iyer Ravi, Boon Huan Tan, Richard J. Sugrue

**Affiliations:** School of Biological Sciences, Nanyang Technological University, 60 Nanyang Drive, Singapore, 637551 Singapore; Detection and Diagnostics Laboratory, DSO National Laboratories, 27 Medical Drive, Singapore, 117510 Singapore

## Abstract

**Background:**

During respiratory syncytial virus (RSV) infection filamentous virus particles are formed on the cell surface. Although the virus infectivity remains cell-associated, low levels of cell-free virus is detected during advanced infection. It is currently unclear if this cell-free virus infectivity is due to a low-efficiency specific cell-release mechanism, or if it arises due to mechanical breakage following virus-induced cell damage at the advanced stage of infection. Understanding the origin of this cell-free virus is a prerequisite for understanding the mechanism of RSV transmission in permissive cells. In this study we describe a detailed examination of RSV transmission in permissive HEp2 cell monolayers.

**Methods:**

HEp2 cell monolayers were infected with RSV using a multiplicity of infection of 0.0002, and the course of infection monitored over 5 days. The progression of the virus infection within the cell monolayers was performed using bright-field microscopy to visualise the cell monolayer and immunofluorescence microscopy to detect virus-infected cells. The cell-associated and cell-free virus infectivity were determined by virus plaque assay, and the virus-induced cell cytotoxicity determined by measuring cell membrane permeability and cellular DNA fragmentation.

**Results:**

At 2 days-post infection (dpi), large clusters of virus-infected cells could be detected indicating localised transmission in the cell monolayer, and during this stage we failed to detect either cell-free virus or cell cytotoxicity. At 3 dpi the presence of much larger infected cell clusters correlated with the begining of virus-induced changes in cell permeability. The presence of cell-free virus correlated with continued increase in cell permeability and cytotoxicity at 4 and 5 dpi. At 5 dpi extensive cell damage, syncytial formation, and increased cellular DNA fragmentation was noted. However, even at 5 dpi the cell-free virus constituted less than 1 % of the total virus infectivity.

**Conclusions:**

Our data supports a model of RSV transmission that initially involves the localised cell-to-cell spread of virus particles within the HEp2 cell monolayer. However, low levels of cell free-virus infectivity was observed at the advanced stages of infection, which correlated with a general loss in cell monolayer integrity due to virus-induced cytotoxicity.

**Electronic supplementary material:**

The online version of this article (doi:10.1186/s12985-016-0467-9) contains supplementary material, which is available to authorized users.

## Background

Respiratory syncytial virus (RSV) is the most important viral cause of lower respiratory tract infection in young children and neonates, leading to high levels of mortality and morbidity [1]. During RSV replication two distinct virus structures are formed in permissive cells, the inclusion bodies and virus filaments. A ribonucleoprotein (RNP) complex is formed by the viral genomic RNA (vRNA), the nucleocapsid (N) protein, the phosphoprotein (P protein), the M2-1 protein and the large (L) protein [2–4]. These RNPs accumulate within the cytoplasmic inclusion bodies [5], and are therefore sites in the cell where the polymerase complex accumulates. The virus filaments are sites of assembly on the surface of infected cells, and in the virus filaments the RNPs are located beneath a protein layer formed by the matrix protein. The virus fusion (F) and attachment (G) proteins are inserted into the virus envelope that surrounds the virus filaments [6, 7]. Both the inclusion bodies and virus filaments have been detected in infected cells obtained from infected patients, suggesting that they have a clinical relevance [8].

Recent evidence has suggested that virus filament formation is a factor in virus transmission [9], and current research is enhancing our understanding of the cellular processes that lead to RSV filament formation [10]. The involvement of lipid-raft microdomains in virus filament formation has been demonstrated [11–15], and the involvement of the cortical actin network in both the formation of virus filaments and virus transmission is suggested [9, 16–18]. A greater understanding of the virus maturation process and the mechanism of virus transmission should greatly facilitate the development of novel antiviral strategies.

Although virus filaments form on the surface of virus infected-cells, in cell-free virus preparations the virus particles typically exhibit pleomorphic morphologies. These cell-free virus particles can range in size from 0.1 μm up to 1 μm in diameter. The existence of these cell-free virus particles in the tissue culture supernatant of virus-infected cells has suggested the existence of a specific mechanism that mediates the release of virus particles from the surface of infected cells. In this context a recent structured-based approach has described a mechanism of virus release to explain the presence of this pleomorphic virus morphology [19]. However, even in tissue culture cells that are highly permissive to RSV infection most of the virus infectivity remains cell-associated [20]. This suggested that if such a mechanism for virus release exists, it is at best of low efficiency. Several previous studies have suggested that localized cell-to-cell transmission is an important mechanism for the spread of RSV infection in tissue culture cells (e.g. [9, 17]). It is therefore not clear if these cell-free virus particles arise due to a specific release mechanism from the infected cells, or if they originate from a non-specific mechanism due to extensive virus-induced cell damage at the advanced stage of infection. In this study we have described a detailed formal examination of the process of RSV transmission in permissive cells in order to determine the biological relevance of the cell-associated virus in relation in RSV transmission. This has enabled us to test the hypothesis that cell-free virus infectivity is due to virus-induced changes in cell physiology that leads to loss of cell integrity and increased cell toxicity.

## Results

### RSV transmission occurs by localised cell-to-cell transmission in HEp-2 cell monolayers

In this study the process of virus transmission was examined using a low multiplicity of infection (moi) cell model. We had previously observed that using a moi of between 0.1 and 0.01 had resulted in 100 % cells in a HEp2 monolayer being infected within 36 h [9]. Therefore unless specified, in this current analysis all infections were carried out in HEp2 cell monolayers using a moi of 0.0002. This lower moi enabled multiple cycles of virus cell infection in the HEp2 cell monolayers to be examined, thus allowing virus transmission to be visualised. The standard RSV plaque assay uses an agarose overlay on HEp2 cell monolayers to contain the released virus in the form of distinct individual virus plagues. However to examine RSV transmission in the HEp2 cell monolayers in our experimental approach we did not use of an agarose overlay, which allowed us to distinguish cell-to-cell virus transmission and cell-free virus transmission. Our preliminary data had indicated that under our experimental conditions extensive cytopathic effect (CPE) within the cell monolayer and high levels of cell detachment occurred after 5 days post-infection (dpi), therefore all subsequent analyses were not performed beyond 5 dpi.

**Fig. 1:**
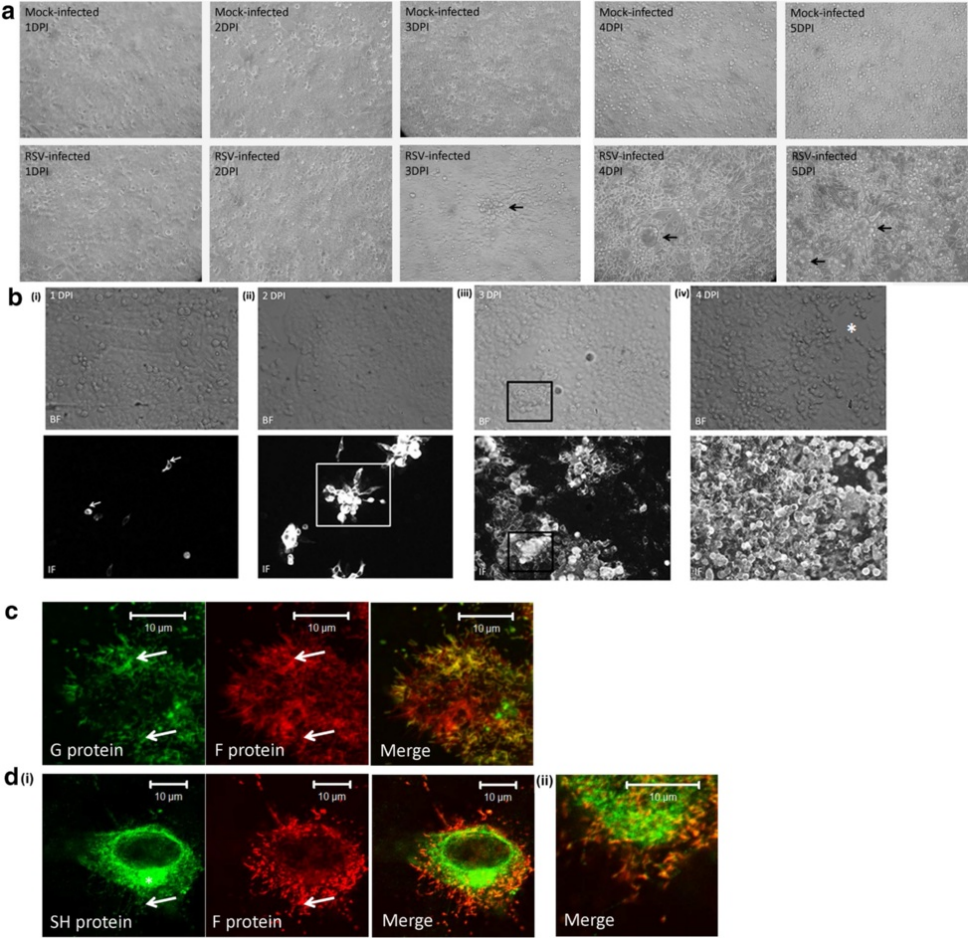
Localized RSV transmission occurs within the HEp2 cell monolayers. HEp2 cell monolayers were either mock-infected or RSV-infected using a multiplicity of infection (moi) of 0.0002. **a** At between 1 and 5 days post-infection (dpi) the monolayers in the tissue culture dish were fixed using 3 % glutaraldehyde and viewed using an inverted light microscope (objective x4 magnification). The virus-induced morphological changes in the monolayer are highlighted (*black arrows*). **b** In a parallel analysis the virus-infected cells were stained using anti-RSV and anti-mouse IgG conjugated to Alexa 488 at (i) 1 dpi, (ii) 2 dpi, (iii) 3 dpi and (iv) 4 dpi. The stained cells were then viewed using fluorescence microscopy (IF) and by bright-field (BF) microscopy (objective x20 magnification). The infected cell cluster (*open white box*), virus-induced morphological changes in the monolayer (*open black box*) and region of the monolayer cleared of cells (*) are highlighted. **c** and **d** Distribution of the F, G and SH virus glycoproteins in the infected cell clusters. HEp2 cell monolayers were infected with RSV using infection moi of 0.01 and 30 h post-infection the cells were fixed and stained using either (**c**) anti-G and anti-F or (**d**) anti-SH and anti-F. **d**(ii) is an enlarged image from (**d**) (i)). The stained cells were viewed using confocal microscopy at an optical plane that allowed imaging of the virus filaments. The virus filaments (*white arrow*) and the typical Golgi staining in the SH stained cells (*) are indicated

HEp2 cell monolayers were either mock-infected or infected with RSV, at between 1 and 5 dpi the cell monolayers were monitored using bright-field (BF) microscopy (Fig. 1a). At between 1 and 2 dpi we observed no visible change in the infected cell monolayers, but by 3 dpi we noted that changes in the cell monolayer had occurred, consistent with the onset of virus-induced CPE. At 4 dpi we noted pronounced morphological changes in the virus-infected cell monolayer, which was accompanied by extensive syncytia formation and cell detachment. Increased syncytia formation and cell detachment (as evidenced by increased numbers of floating cells) was noted at 5 dpi.

In a parallel analysis HEp2 cell monolayers were infected with RSV and between 1 and 4 dpi the monolayers were stained using anti-RSV and anti-mouse IgG-FITC (to detect virus-infected cells). The monolayers were examined by BF microscopy (to detect the cell monolayer) and immunofluorescence (IF) microscopy (to detect the presence of infected cells within the monolayer) (Fig. 1b). At 1 dpi individually infected cells within the cell monolayer could be detected (Fig. 1b (i)), and by 2 dpi the presence of larger infected cell clusters could be detected (approximately 25 ± 5 cells per cluster) (Fig. 1b (ii)). At 3 dpi we noted the presence of fewer but much larger infected cell clusters (>100 cells per cluster) (Fig. 1b (iii)). We also noted that in some instances morphological changes in the cell monolayer within the interior of the infected cell clusters (Fig. 1b (iii)). At 4dpi extensive staining across the cell monolayer was observed, together with anti-RSV-stained syncytia (Fig. 1b (iv)). We also noted that some regions in the cell monolayer were devoid of cells, presumably a consequence of cell-detachment. Therefore the addition of very low levels of initial virus to the monolayer led to the formation of infected cell clusters containing large numbers of infected cells, thus highlighting the extensive localized virus transmission in the cell monolayer by 3 dpi.

The pattern of staining observed using this low moi infection model suggested localized cell-to-cell transmission within the monolayer. On average we observed between 5 and 8 singly infected cells within the cell monolayer at 24 hpi, and by 48 hpi we observed a similar number of large infected cell clusters. It would be expected that if a mechanism of efficient virus release occurred then we would expect the spread of cell-free virus particles across the cell monolayer (e.g. via Brownian motion) in the tissue culture medium. This would be expected to give rise to a larger number of smaller and randomly distributed anti-RSV stained cell within the monolayer; rather than the formation of localized clusters of infected cells that we observe. Since infected cell clusters in the anti-RSV stained monolayer could be detected by 2 dpi, in a similar analysis we examined the distribution of individual virus structural proteins. Cells were stained using antibodies against the G protein, F protein, N protein, P protein, M2-1 protein, M protein and SH protein at 2 dpi and examined using IF microscopy. In all cases infected cell clusters were observed at 2 dpi (Additional file 1: Figure S1A) similar to that observed with the anti-RSV staining. This indicated that the appearance of the infected cells clusters was not due to the use of a specific immunological reagent.

The appearance of virus filaments on anti-G stained cells within the cell monolayer was evident at higher magnification (Additional file 1: Figure S1B), and this was confirmed using confocal microscopy. The F, G and SH virus surface membrane proteins have been detected in the purified virus particles [21], and these would be expected to be present in virus filaments. We therefore examined their staining distribution to confirm their presence in the HEp2 cell monolayer. The HEp2 cell monolayers were infected with RSV using a moi of 0.01, and at 24 hpi the cells were co-stained using both anti-G and anti-F (Fig. 1c) and anti-SH and anti-F (Fig. 1d). Examination of the co-stained cells using confocal microscopy revealed the prominent filamentous F and G staining patterns that have been reported previously [7], and which indicated the presence of virus filaments in the cell monolayer. Although we observed the prominent anti-SH Golgi-type staining pattern [22], we also noted a filamentous anti-F and anti-SH co-staining pattern. This confirmed the presence of the SH protein in the virus filaments [21, 22], and collectively these data confirmed the presence of virus filaments in the infected cell monolayers.

**Fig. 2:**
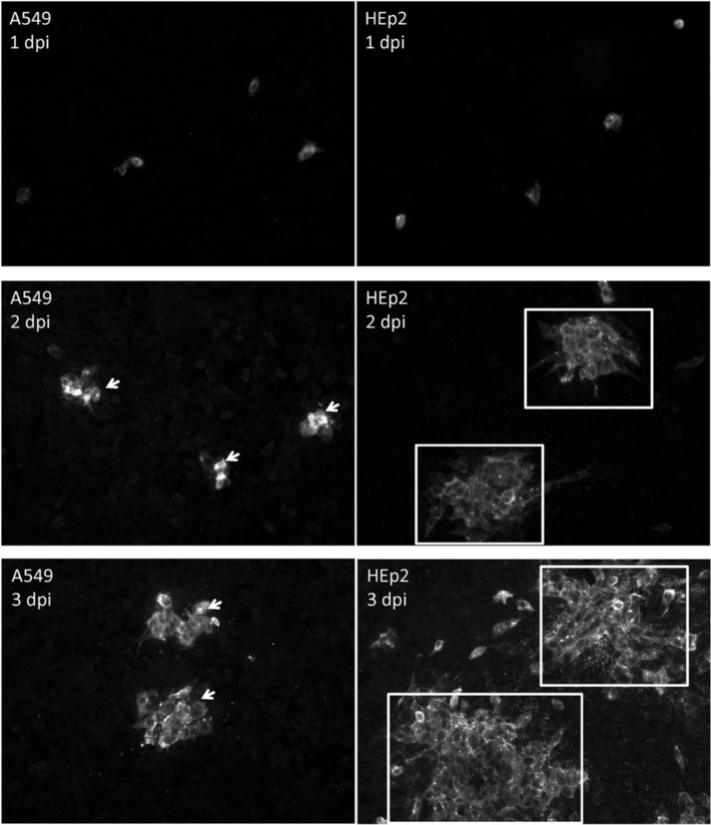
Localized RSV transmission occurs with the A549 cell monolayers. A549 and HEp2 cell monolayers were infected with RSV using a multiplicity of infection (moi) of 0.0002 and at between 1 and 3 days post-infection (dpi) the virus-infected cells were stained using anti-RSV and anti-mouse IgG conjugated to Alexa 488 and viewed using fluorescence microscopy (objective x20 magnification). The infected cell clusters in the A549 (*white arrows*) and HEp2 (*open white box*) cell monolayers are highlighted

In a similar analysis we compared the transmission of RSV in both HEp2 and A549 cells. Cell monolayers were infected with RSV and at 1, 2 and 3 dpi the cells were stained with anti-RSV and examined by IF microscopy. At 1 dpi similar numbers of single infected cells within each cell monolayer was detected, and a similar number of infected cell clusters could be detected in each monolayers at 2 and 3 dpi (Fig. 2). This indicated that the formation of infected cell clusters was not specific to HEp2 cells; consistent with our previous observations [9]. However, at both 2 and 3 dpi the infected clusters in the HEp2 cell monolayers contained 37.2 ± 13 cells per cluster, while in A459 cells we noted 8.5 ± 5 cells per cluster. This suggested that the RSV transmitted more efficiently in HEp2 cells monolayers compared to A549 cell monolayers. MDCK cells are also permissible for RSV infection and we examined RSV transmission in MDCK cell monolayers grown on either glass or on Transwell™ membranes (Additional file 2: Figure S2). The cells were infected with RSV and at between 1 and 5 dpi the monolayers were stained using anti-RSV and examined using IF microscopy. Although the infected cell clusters appeared to be smaller in the MDCK cell monolayers grown on the Transwell™ membranes when compared to the MDCK cell monolayers grown on a glass substrate, similar infected cell clusters could were also detected under both culturing conditions at 2 and 3 dpi.

The microscopic examination described above was consistent with occurrence of two phases of virus infection in the low moi infection model. At an early stage (up to 3 dpi) the formation of infected cell clusters was observed that was consistent with localised cell-to-cell virus transmission. The presence of the virus filaments suggested that this localized virus transmission occurs via these virus-induced structures. Interestingly, this localized virus transmission was not associated with obvious signs of RSV-induced CPE in the cell monolayer. Morphological changes in the cell monolayer became apparent at 3 dpi, consistent with the start of virus-induced CPE. These changes in the cell monolayer became more extensive at 4 and 5 dpi, with extensive CPE and cell detachment being evident. These observations suggested that although the process of virus transmission eventually gives rise to syncytial formation, the processes of virus transmission and syncytial formation are two distinct processes.

### Localised virus transmission is accompanied by localized changes in F-actin

**Fig. 3:**
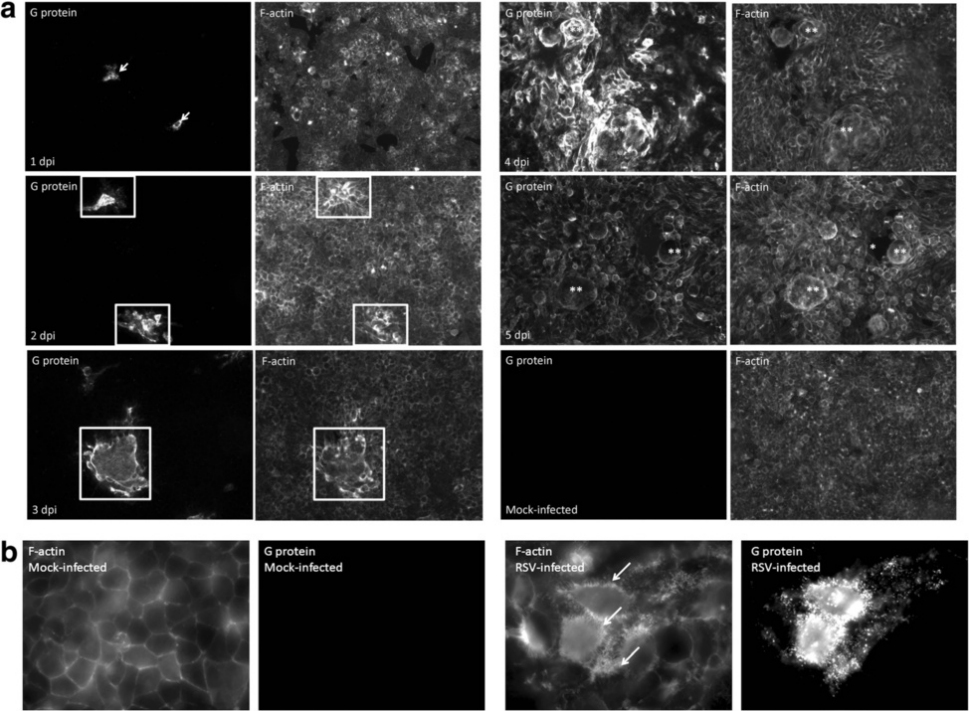
RSV-infection induces localized changes in the F-actin distribution in HEp2 cell monolayers. **a** HEp2 cell monolayers were infected with RSV using a multiplicity of infection of 0.0002 and at between 1 and 5 days post-infection (dpi) the cells were co-stained using anti-G and phalloidin-FITC (to detect F-actin). The mock-infected sample was stained at 5 dpi. The stained cell monolayers were viewed by immunofluorescence microscopy (objective x20 magnification). Individual infected cells at 1 dpi (*white arrows*), infected cell clusters (*open white box*), cell detachment (*) and syncytia (**) are indicated. **b** Virus-induced increased phalloidin-FITC staining at the intercellular junctions. Mock and virus-infected cells at 2 dpi were stained with anti-G and phalloidin-FITC. The increased phalloidin-FITC staining in the virus infected cells is highlighted (*white arrows*). The stained cell monolayers were viewed by immunofluorescence microscopy (objective 100x magnification)

The involvement of the F-actin remodeling in both the formation of virus filaments and mediating virus transmission has been suggested [9, 16–19]. Previous studies have also shown that F-actin polymerisation inhibitor cytochalasin D is able to both inhibit virus filament formation, and prevent virus cell-to-cell transmission [9]. RSV transmission was examined in anti-G and phalloidin-FITC (to detect F-actin) stained cell monolayers at between 1 and 5 dpi using IF microscopy (Fig. 3a). At low magnification the presence of infected cell clusters were detected at 2dpi, and increased phalloidin-FITC staining at the inter-cellular contacts (i.e. cell junctions) between the individual cells within the cell clusters was observed (Fig. 3a). At higher magnification virus-induced changes in the F-actin staining pattern within the infected cell clusters and the increased F-actin staining at the cell junctions was more apparent (Fig. 3b). This suggested localized virus-induced changes in the F-actin distribution during virus transmission, and was consistent with a role for F-actin in mediating virus filament formation and RSV transmission as described previously [9, 17]. The change in F-actin distribution became more apparent at 3 dpi as the number of infected cell clusters within the infected cell clusters increased in size (Additional file 3: Figure S3A). However, at 3 dpi the intercellular phalloidin-FITC staining between individual cells within some of the infected cell clusters became less defined (Fig. 3a; Additional file 3: Figure S3B). In these cases, although increased F-actin staining was noted at the leading edge of these infected cell clusters, the F-actin staining pattern between individual cells within these clusters could not be distinguished. This suggested intercellular membrane fusion between the individual cells had occurred, consistent with the start of syncytia formation. At 4 and 5 dpi the appearance of large phalloidin-FITC stained syncytia indicated further intercellular membrane fusion and syncytia formation at the later stages of infection. In contrast, an intact cell monolayer and the presence of clearly defined phalloidin-FITC-stained intercellular junctions was observed in mock-infected cells at 5 dpi (Fig. 3a).

**Fig. 4:**
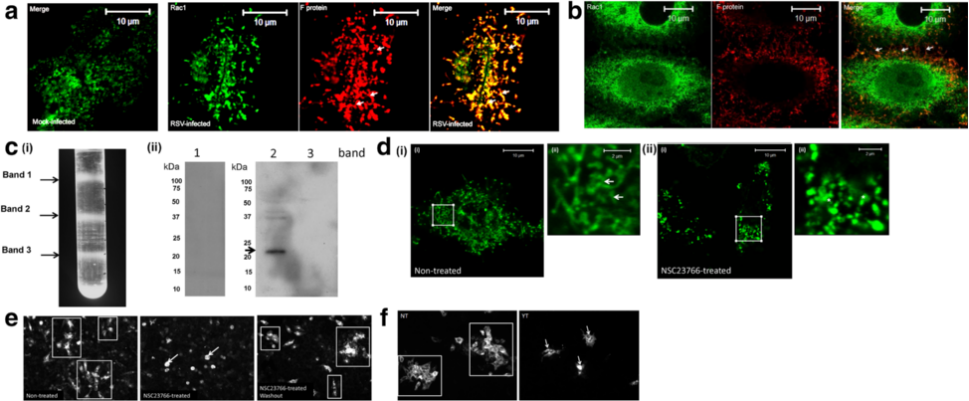
The rac-1 and rho A kinases are required for localised cell-to-cell transmission in the HEp2 cell monolayer. **a** Mock and RSV-infected cells (multiplicity of infection (moi) of 0.1) were stained using anti-F and anti-rac-1 at 24 h post-infection (hpi) and imaged by confocal microscopy (objective x100 magnification). Only the merged image is shown in the mock-infected cells. **b** Co-stained cells showing virus filaments bridging two cells. In all cases the virus filaments are highlighted (*white arrows*). **c** Virus particles were dissociated from infected cells and infectious virus particles isolated using discontinuous sucrose gradient centrifugation as described previously [21]. **c** (i) A representative gradient showing the locations of the fractions at the 20-35 % sucrose (band-1), the 35-45 % sucrose (band-2) and 45-55%sucrose (band-3) interfaces. (ii) Each fraction was examined by immunoblotting using anti-rac-1. A protein band corresponding in size to the rac-1 protein is highlighted (*black arrow*). **d** and **e**The rac-1 inhibitor NSC23766 prevents virus filament formation and virus transmission. **d** RSV-infected HEp2 cells (moi = 0.1) were either non-treated or NSC23766-treated and the cells stained using anti-G. The virus filaments in non-treated (*white arrows*) and punctuate staining pattern in NSC23766-treated cells (*) are highlighted. In each case (ii) is an enlarged image of the region highlighted by the white box in (i). **e** rac-1 activity is required for cell-to-cell transmission. HEp2 cell monolayer were infected using a moi of 0.0002 and at 5 hpi the cell were either non-treated or NSC23766 treated. At 18 hpi the inhibitor was either removed (washout) or maintained. At 36 hpi the cells were fixed and stained using anti-RSV. The cells were imaged using immunofluorescence microscopy (objective x20 magnification). The infected cell clusters in the NT cells (*open white box*) and smaller clusters in the NSC23766-treated cells (*white arrows*) are indicated. **f** HEp2 cell monolayers were infected with RSV using a moi of 0.0002 and at 18 hpi the cells were either non-treated (NT) or treated with Y-27632 (YT). At 48 hpi the cells were fixed and stained using anti-RSV and imaged using immunofluorescence microscopy (objective x20 magnification). The infected cell clusters in the NT cells (*open white box*) and smaller clusters in the YT cells (*white arrows*) are indicated

Cellular actin structures are regulated via several different rho GTPase proteins and their down-stream effectors [23, 24]. In this context the rac-1 kinase has previously been shown to play a role in RSV morphogenesis [19, 25], and we therefore examined if the rac-1 protein also played a role in mediating localised virus transmission in the cell monolayer. Cells were infected with RSV using a moi of 0.1, and at 24 hpi the cells were stained using anti-rac-1 and anti-F and examined using confocal microscopy. We noted co-staining of both proteins within the virus filaments (Fig. 4a), and the presence of co-stained virus filaments extending between cells (Fig. 4b). To confirm the presence of the rac-1 protein in the virus filaments, the virus filaments were mechanically released from the infected cells and isolated as described previously [21] using discontinuous sucrose gradient centrifugation (20 % (w/v) sucrose, 35 % (w/v) sucrose, 45 % (w/v) sucrose and 60 % (w/v) sucrose). The material at the 20-35 % (w/v) sucrose (band-1 fraction), 35-45 % (w/v) sucrose (band-2 fraction) and 45–60 % (w/v) sucrose (band-3 fraction) interfaces were harvested (Fig. 4c(i)), and HEp2 cell monolayers were challenged with each fraction to confirm that virus infectivity was restricted to the band-2 (Additional file 4: Figure S4) [21]. Examination of the three fractions by immunoblotting with anti-rac-1 showed the presence of rac-1 protein only in the band-2 fraction (Fig. 4c(ii)). Although several specific host cell proteins are incorporated into filaments [21], collectively the biochemical and microscopic analyses indicated the presence of rac-1 protein at the site of virus transmission.

The small molecule NSC 23766 is an established rac-1 inhibiter that blocks rac-1 activation [26]. We confirmed our earlier observation [19] that virus filament formation was inhibited by NSC23766 treatment (Fig. 4d). However, we also used NSC23766 treatment in this study to determine if rac-1 activation also played a role in the localized virus transmission in the HEp2 cell monolayer. HEp2 cell monolayers were infected and at 5 hpi the cells were either non-treated or treated with NSC23766. At 18 hpi the inhibitor was either maintained or removed and the cell stained with anti-RSV at 30 hpi (Fig. 4e). We noted in the non-treated cell monolayers the presence of relatively large infected cells clusters (14 ± 3 cells per cluster); while in NSC23766-treated cells only single infected cells were detected. In cells where the inhibitor was removed a degree of cell-to-cell transmission was restored. This indicated that NSC23766 treatment impaired localized virus transmission in the monolayer and provided evidence that the rac-1 protein played a role in mediating the localized virus transmission.

Since the rhoA kinase has been shown to play a role in virus filament formation and in the formation of syncytium [27] we therefore examined the effect of the rhoA kinase inhibitor Y-27632 on virus transmission (Fig. 4f). The HEp2 cell monolayers were infected with RSV and at 18 hpi the cells were either non-treated or Y-27632-treated. At 48 hpi the cells were stained using anti-RSV and examined using IF microscopy. While efficient localized virus transmission was observed in the non-treated cells, we noted significantly smaller cluster of infected cells in Y-27632-treated cells. This indicated impaired virus transmission, suggesting a role for the rhoA kinase in mediating virus transmission in the HEp2 cell monolayer. Pretreatment of cell with Y-27632 did not inhibit RSV infection (Ravi and Sugrue, unpublished observations), consistent with previous observations [27].

### Virus-induced changes in cell signaling occur in the infected cell clusters at 3 dpi

**Fig. 5:**
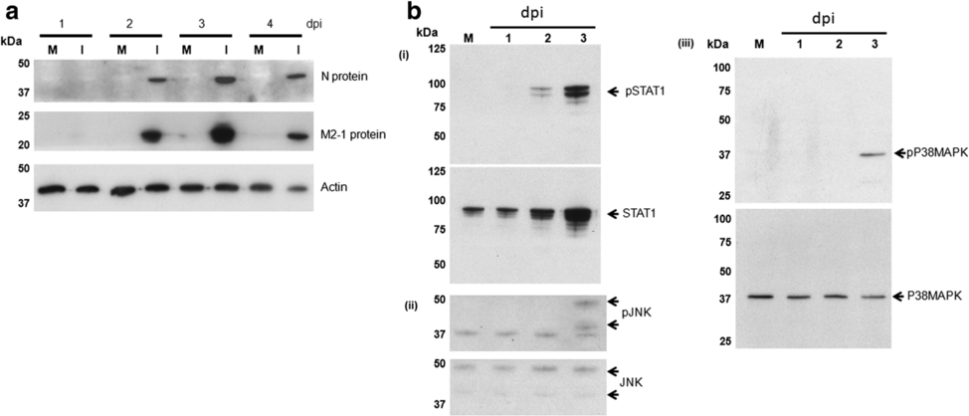
Virus infection induces cell signaling pathways associated with cell death at 3 dpi. **a** HEp2 cell monolayers were either mock-infected (M) or infected with RSV using a multiplicity of infection of 0.0002. At between 1 and 4 days post-infection (dpi) cell lysates were prepared and the presence of the N and M2-1 proteins detected by immunoblotting. Actin is the loading control. **b** The presence of (i) STAT1 and pSTAT1, (ii) JNK and pJNK and (iii) MAPKp38 and pMAPKp38 were detected in the RSV-infected cell lysates by immunoblotting with the relevant antibody at 1, 2 and 3 dpi. In this case the infected cells were compared with the mock-infected cell lysate harvested at 3 dpi

The RSV N and M2-1 protein are present in mature virus particles, and the N protein is one of the most abundant virus structural proteins present within the mature infectious virus particles. The progress of virus infection in the HEp2 cell monolayer was therefore examined by detecting the presence of the N and M2-1 proteins in the infected cell monolayers. Cell lysates were prepared in mock-infected and RSV-infected cell lysates at between 1 and 4 dpi and immunoblotted using anti-N and anti-M2-1 (Fig. 5a). At 1 dpi we failed to detect the presence of either virus protein, suggesting that the levels of expressed virus protein were below the level of detection using our experiential conditions. However, by 2 dpi the presence of the N and M2-1 proteins were readily detected, and this was followed by a further increase in the levels of both virus proteins at 3 dpi. At 4 dpi a decrease in the M2-1 and N protein levels was noted (when compared to that at 3 dpi), together with a reduction in the actin protein levels. We reasoned that this was due to the loss of infected cells from the monolayer by cell detachment that we observed at 4dpi.

Induction of type 1 interferon expression leads to the activation of STAT1, and several studies have demonstrated that type 1 interferon signaling (via the JAK/STAT pathway) is able to activate several cellular kinases (e.g. the MAPKp38 and JNK signaling pathways) that play a role in initiating the molecular processes that eventually leads to cell death (reviewed in [28]). We therefore examined the cell lysates by immunoblotting using antibodies that recognize STAT1, JNK and MAPKp38 and the activated phosphorylated forms of these proteins, namely pSTAT1, pJNK and pMAPKp38. Due to the cell loss at 4 dpi, this analysis was performed using the cell lysates that were harvested at between 1 and 3 dpi (Fig. 5b).

**Fig. 6:**
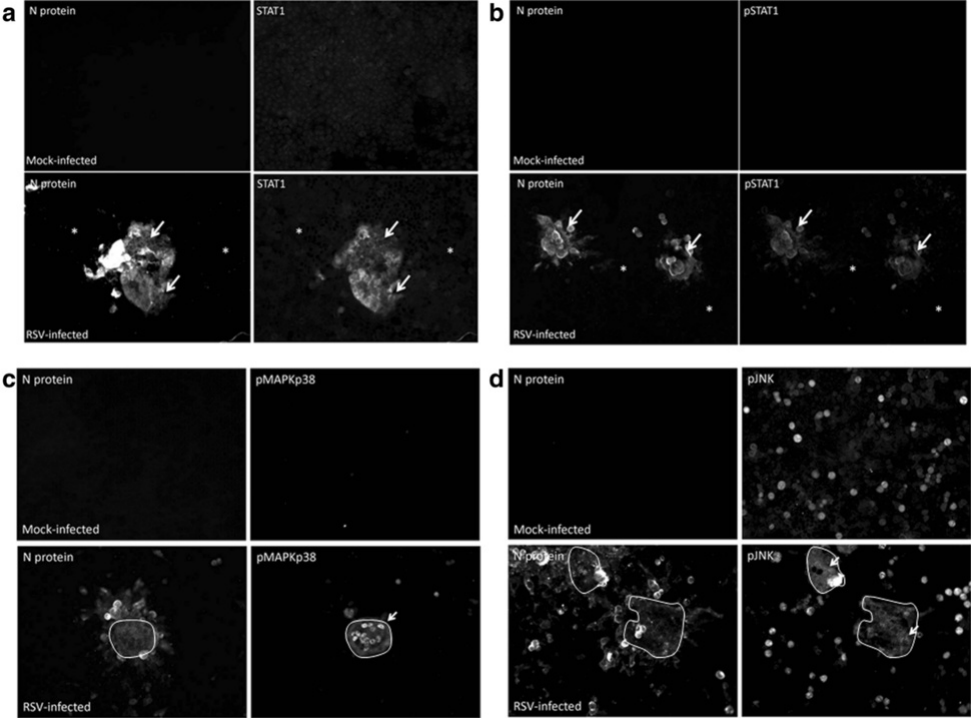
Distribution of STAT1, pSTAT1, pMAPKp38 and pJNK in the RSV-infected monolayers. HEp2 cell monolayers were mock-infected or infected with RSV using a multiplicity of infection of 0.0002. **a** and **b** At 2 days post-infection (dpi) the cells were stained using anti-N and either (**a**) anti-STAT1 or (**b**) anti-pSTAT1. The cells were viewed by IF microscopy (objective x20 magnification). The STAT1 and pSTAT1 regions in the cell monolayer are highlighted (*white arrow*), and the region of the monolayer containing non-infected cells is indicated (*) (**c** and **d**) HEp2 cell monolayers were mock-infected or infected with RSV using a multiplicity of infection of 0.0002. At 3 dpi the cells were stained using anti-N and either (**c**) anti-pMAPKp38 or (**d**) anti-pJNK. The cells were viewed by fluorescence microscopy (objective x20 magnification). The pMAPKp38 and pJNK regions in the cell monolayer are indicated (*white arrow*). The region of pMAPKp38 and pJNK staining in each image is demarcated (*white outline*)

At 1 dpi similar total levels of STAT1 protein expression could be detected in both the mock-infected and RSV-infected cell lysates (Fig. 5b(i)), however by 2 dpi a small increase in STAT1 protein levels in the infected cells correlated with the appearance of low pSTAT1 levels. At 3 dpi a further increase in both STAT1 and pSTAT1 protein levels in the infected cells indicated that virus-induced interferon signaling at between 2 and 3 dpi. The delay in interferon signaling with respect to virus transmission was consistent with recent observations that interferon signaling occurs relatively late in the RSV replication cycle [29]. Furthermore, analysis of RSV-infected cells by IF microscopy at 48 hpi showed that the increased STAT1 staining (Fig. 6a) and pSTAT1 staining (Fig. 6b) was restricted to the infected cell clusters, and was not detected in the non-infected cells that surrounded the infected cell clusters.

Similar JNK and MAPKp38 protein levels were detected in mock-infected and RSV-infected cell monolayers at between 1 and 3 dpi (Fig. 5b (ii) and (iii)). However, the presence of pJNK and pMAPKp38 were detected at 3 dpi, and indicated that virus-induced activation of the JNK and MAPKp38 signaling pathways at 3 dpi. Analysis of RSV-infected cells by IF microscopy at 3 dpi showed that the pMAPKp38 (Fig. 6c) and pJNK (Fig. 6d) staining was largely confined to the central regions of the infected cell clusters, and that staining with either antibody was not detected at the leading edge of the infected cell clusters. This indicated that these pathways were activated in cells showing advanced infection, but were not activated in newly infected cells at the leading edge of the infected cells clusters. This was consistent with delayed activation of the JNK and MAPKp38 signaling pathways with respect to virus transmission. As expected, anti-pMAPKp38 and anti-pJNK staining was not detected either in the non-infected cells that surrounded the infected cells clusters, or in the mock-infected cell monolayers.

### The presence of cell-free virus infectivity correlates with increased cell membrane permeability and cell cytotoxicity

**Fig. 7:**
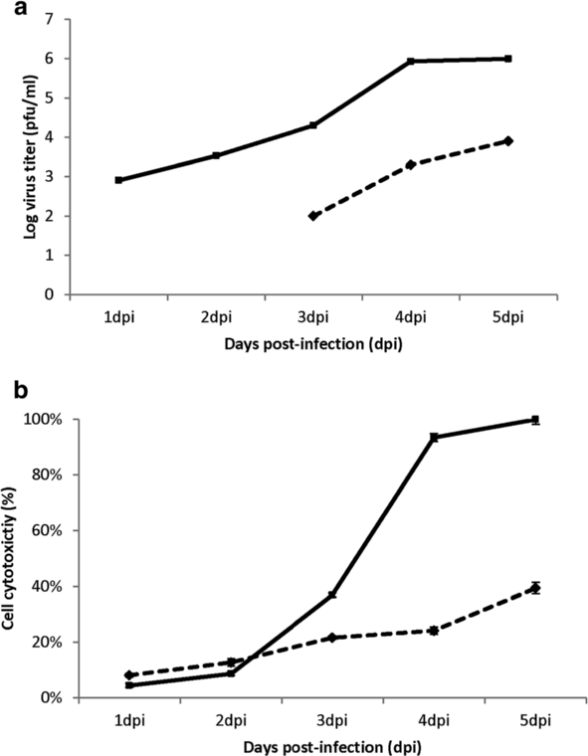
The presence of cell-free virus particles occurs late in infection and correlates with increased cell permeability and cytotoxicity. HEp2 cell monolayers were infected with RSV using an multiplicity of infection of 0.0002 and (**a**) between 1 and 5 days post-infection (dpi) the cell-associated virus infectivity (solid line) and the cell-free virus infectivity (broken line) in the tissue culture supernatant was measured by plaque assay. Average values (duplicates) for the infectivity measurements are shown and for all times the standard error for each of the infectivity measurements was ≤ 10 %. Representative data obtained from one experiment is shown. **b** Lactate dehydrogenase (LDH) release from RSV-infected HEp2 cells. HEp2 cell monolayers were either mock-infected (*broken line*) or RSV-infected (*solid line*) using a multiplicity of infection of 0.0002 and at between 1 and 5 dpi the levels of LDH was measured in the tissue culture supernatant. The values are average values of triplicate measurements at each time point. Representative data from one experiment is shown

The imaging analysis described above suggested intercellular virus transmission involving cell-associated virus, followed by extensive CPE within the monolayer after 3 dpi. We therefore examined the relative levels of cell-free and cell-associated virus infectivity between 1 and 5 dpi. HEp2 cells were infected with RSV using a moi of 0.0002 and at between 1 and 5 dpi the cell-associated (CA) virus infectivity (i.e. the cell fraction) and cell-free (CF) virus infectivity (i.e. in the clarified tissue culture supernatant) was measured at each time of infection (Fig. 7a). By 1 dpi we could detect CA-virus infectivity, and a temporal increase in CA-virus infectivity was noted over the five days of infection, which appeared to plateau at between 4 and 5 dpi. In contrast the CF-virus infectivity was not detected at either 1 or 2 dpi, despite the appearance of CA-virus infectivity at these times of infection, and the presence of large infected cell clusters in the HEP2 cell monolayers at 2dpi. At 3 dpi the appearance of low levels of CF-virus could be detected, and there was a subsequent increase in the levels of CF-virus at 4 and 5 dpi. However, at all times of infection the CF-virus infectivity contributed less than 1 % of the total virus infectivity in the infected cell monolayer. This indicated that the virus infectivity was largely cell-associated throughout the infection, even under conditions where extensive CPE was evident (e.g. at 5 dpi).

The presence of the CF-virus at 3 dpi correlated with morphological changes in the cell monolayer, and we examined if the appearance of the CF-virus correlated with loss of cell viability. We used the established lactate dehydrogenase (LDH) release cytotoxicity assay which measures the levels of LDH released from cells following the loss of membrane integrity. The LDH levels in the tissue culture supernatant of mock-infected cells and cells infected with RSV using a moi of 0.0002 at between 1 and 5 dpi (Fig. 7b). At 1 and 2 dpi we observed similar LDH levels in the tissue culture supernatant of both mock-infected and RSV-infected cells. This suggested no significant levels of cell cytotoxicity or loss of membrane integrity had occurred during the first two days of infection. However, by 3 dpi we noted a 10 % increase in LDH levels in the tissue culture supernatant of virus-infected cells compared to that in mock-infected cells. A further increase in LDH levels in the tissue culture medium of virus-infected cells compared to that in mock-infected cells was detected at 4 and 5 dpi. Collectively, these data indicated the start of virus-induced cell membrane permeability at 3 dpi, and that this virus-induced cell membrane permeability increased dramatically at 4 and 5 dpi.

**Fig. 8:**
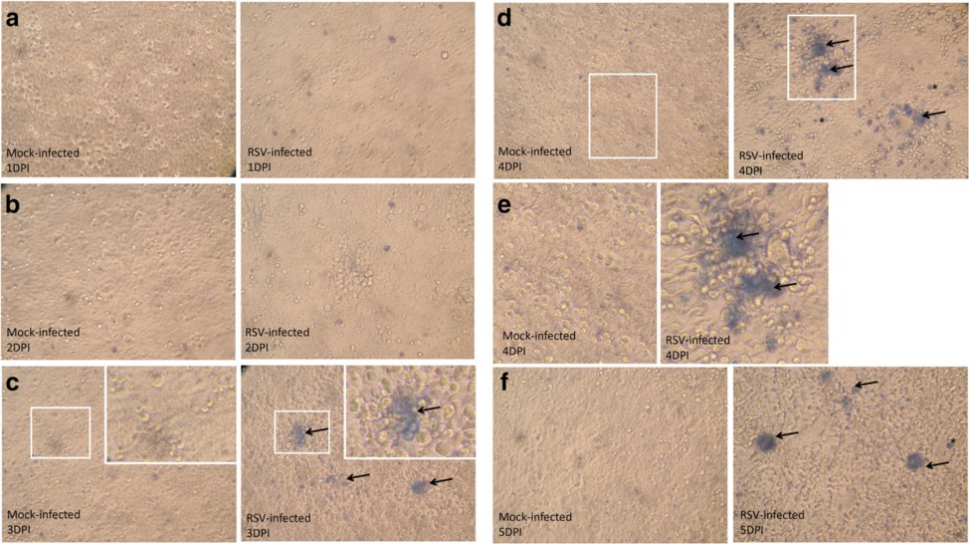
Trypan blue staining is detected in the HEp2 cell monolayers. HEp2 cell monolayers were either mock-infected or RSV-infected using an multiplicity of infection of 0.0002 and (**a**-**f**) at between 1 and 5 days post-infection (dpi) the monolayers were stained using trypan blue and imaged using an inverted light microscope (objective x10 magnification). The regions of the cell monolayer showing trypan blue staining is highlighted (*black arrows*). In plate (**c**) selected regions in the monolayer (indicated by *open white box*) are enlarged in the inset. Plate (**e**) shows enlarged images from the region indicated in plate (**d**) (indicated by *open white box*)

The presence of CF-virus correlated with increased membrane permeability, suggesting that its presence may arise due to changes in cell membrane integrity and increased cytotoxicity. Trypan Blue (TB) is a vital stain that preferentially stains dead cells, where the cell membrane becomes permeable to TB uptake. We therefore used TB staining to visualize the cytotoxicity within the cell monolayer following virus infection. Although in essence the TB uptake assay measures the same parameter as the LDH released assay (i.e. cell permeability), the TB assay allows us to visualize the cells within the monolayer that exhibit this altered permeability. At 1 and 2 dpi we failed to detect significant TB staining within the mock-infected and virus-infected cell monolayers, indicating no significant loss of membrane permeability (Additional file 5: Figure S5). However by 3 dpi TB staining in the virus-infected cell monolayer was noted, which increased at 4 and 5 dpi. Similar TB-staining was not detected in mock-infected cells between 1 and 5 dpi, indicating that the TB staining in the infected monolayers from 3 dpi was due to virus infection. A more detailed analysis of the TB-stained infected cell monolayer indicated that the TB staining was primarily associated with regions in the cell monolayer undergoing virus-induced morphological changes (Fig. 8). Furthermore, the syncytia, which are a common diagnostic feature of RSV infection, exhibited intense TB-staining. These data confirmed the LDH release assay data, indicating that the appearance of the CF-virus correlated with the beginning of altered virus-induced membrane integrity and loss of cell viability at 3 dpi.

### DNA fragmentation in cell monolayers occurs at the latter stages of RSV infection

**Fig. 9:**
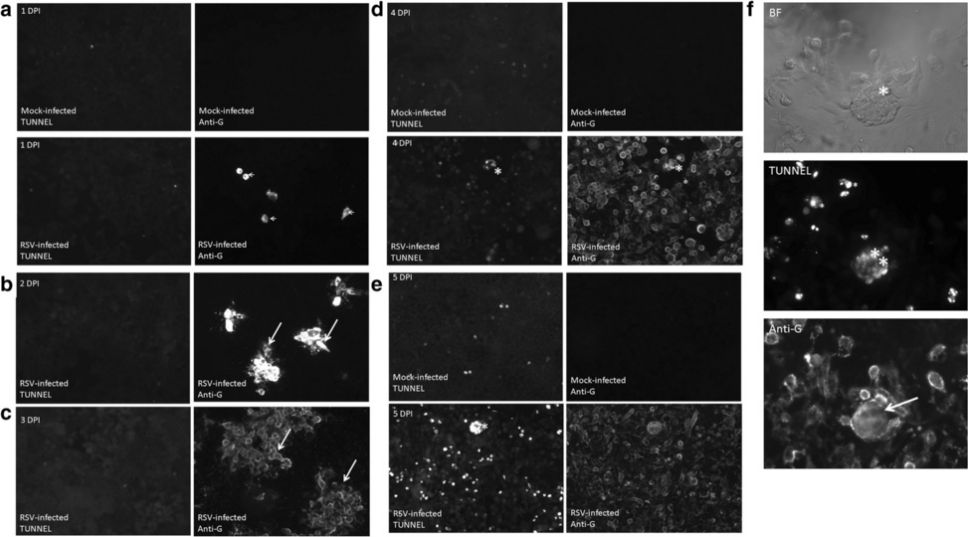
TUNEL assay of virus-infected HEp2 cell monolayers. HEp2 cell monolayers were either mock-infected or infected with RSV using a multiplicity of infection of 0.0002. At (**a**) 1 day post infection (dpi), (**b**) 2 dpi, (**c**) 3 dpi, (**d**) 4 dpi and (**e**) 5 dpi the monolayers were co-stained using anti-G and TUNEL. The stained cells were then viewed using fluorescence microscopy. The anti-G infected cell clusters (*white arrows*) and the increased TUNEL staining (*) are indicated (objective x20 magnification). (**f**) Enlarged image showing virus-induced syncytia viewed using bight field (BF), TUNEL staining, and anti-G staining at 5 dpi. The syncytia (*), TUNEL staining (**) and anti-G staining (*white arrow*) are highlighted. Mock infected monolayers are shown at 1, 4 and 5 dpi

The TUNEL staining assay detects the presence of DNA fragmentation in the cell nucleus which occurs during the later stages of cell death (e.g. during necrosis or apoptosis). Cell death would be an expected consequence of altered membrane integrity and TUNEL staining was performed on cells monolayers at between 1 and 5 dpi. TUNEL staining of the HEp2 cell monolayers allowed us to detect areas within the cell monolayer where the cells showed evidence of cell death. HEp2 cell monolayers were either mock-infected or infected with RSV and at between 1 and 5 dpi the cell monolayers were co-stained using anti-G and TUNEL (Fig. 9). In mock-infected cells we failed to detect the presence of anti-G staining, and a low level of sporadic TUNEL staining throughout the cell monolayer (that slightly increased over the 5 days) was noted. In the RSV-infected cell monolayer single anti-G stained cells at 1 dpi were detected (Fig. 9a), and anti-G stained infected cell clusters were detected at 2dpi (Fig. 9b). Although the infected cells clusters increased in size by 3 dpi (Fig. 9c), TUNEL staining in the virus-infected monolayers at between 1 and 3 dpi was not observed. This indicated that although several cycles of virus replication in the monolayer had occurred, this was not sufficient to cause DNA fragmentation at this time of infection. This is consistent with several reports indicating the ability of the virus to prevent apoptosis in infected cells by a PI3K-depedant signaling pathway involving the down-stream effectors AKT and GSK [30].

At 4 and 5 dpi all cells within the virus-infected cell monolayer showed extensive anti-G staining (Fig. 9d and e), and CPE and syncytia formation was also evident. At 4 dpi the TUNEL staining was restricted to the syncytia (Fig. 9d), while at 5 dpi the TUNEL stained syncytia was accompanied by individual TUNEL stained cells throughout the virus-infected cell monolayer (Fig. 9d and f). This accounted for an approximate 500 % increase in the numbers of individual TUNEL-stained cells compared with that in mock-infected cells at 5 dpi. This indicated that increased virus-induced cell death in the monolayer occurred at these later stages of infection.

## Discussion

We have used a low moi cell infection model to examine RSV transmission in HEp2 cell monolayers. Using this experimental approach we identified two distinct temporal phases in virus transmission. An early stage in the virus infection occurred at between 0 and 3 dpi that involved efficient localized cell-to-cell virus transmission, and no significant adverse effect on cell viability was apparent. This was followed by a later stage (at between 3 and 5 dpi) that was associated with profound changes in cell physiology, loss of cell viability, and the presence of low levels virus infectivity in the tissue culture supernatant.

In the early phase the virus infectivity in the cell monolayer was restricted to clusters of infected cells, consistent with that localized virus transmission within the cell monolayer. A direct role for F-actin in the formation of virus particles and RSV transmission has been suggested by several studies [9, 16, 17, 19]. Furthermore, evidence that actin remodeling mediates localised virus transmission in the cell monolayer is suggested by the observation that rac-1 and rhoA kinase inhibitors block virus particle assembly [19, 25, 27, 31]. In this current study inhibiting rac-1 or rhoA kinase also prevented the localised virus transmission in the cell monolayer. Collectively these earlier studies support a model of virus transmission in which F-actin plays a direct role in both virus morphogenesis and localized virus transmission, leading to the formation of the infected cell clusters. Although we have used the highly permissive HEp2 cell line in this study, we have also observed this time-dependent virus transmission in cell monolayers using other RSV-permissive cell types (e.g. A549 and MDCK cells). Similarly, the presence of infected cell clusters was also observed using other fixation protocols (Ravi Iyer and Sugrue, unpublished observations).

Significant levels of cell cytotoxicity was not detected during this early stage of virus infection, indicating that during the early stage of virus transmission there was no apparent deleterious effect on cell viability, and that the process of virus cell-to-cell transmission per se did not directly induce cell death. Virus-induced changes in cell viability became apparent from 3 dpi and correlated with the presence of cell-free virus infectivity, suggesting that the cell-free virus particles may arise due to changes in cell membrane integrity and cell viability. However, even at 5 dpi when extensive cytotoxicity and syncytia formation was observed, the CF-virus particles only contributed between 0.5 and 0.8 % of the total virus infectivity. This was consistent with the low level of cell-free virus infectivity due to indirect effects of virus-induced cell damage. This would be expected to cause the mechanical detachment of virus filaments from the infected cells into the surrounding milieu. This further suggests that the CF-virus particles that are detected are an indirect consequence of widespread cell-to-cell virus transmission that leads to extensive CPE.

In the clinical context, viruses that are transmitted by direct cell-to-cell contact would have advantages over a predominantly cell-free virus state, for example evasion of the host immune response by HIV-1 during cell-to-cell transmission via the immune synapse [32, 33]. In the case of respiratory viruses the direct cell-to-cell transmission during the initial stages of infection could avoid the release of the virus particles into a potentially hostile environment of the mucus-coated upper respiratory tract during this key stage in the infection process, which could potentially impair virus infection and transmission [34]. Interestingly, we have observed a similar spread of infection when non-tissue culture adapted RSV clinical strains were cultured in HEp2 cell monolayers [35]. Although the spread of infection of the clinical stains was significantly slower than that of the RSV A2 isolate, the infection of the clinical strains in HEp2 cell monolayers was manifested by localized cell-to-cell virus transmission in the cell monolayer. This preceded the extensive CPE (e.g. typically appearing as syncythia) that we observed later in the cell monolayers infected with these clinical strains (usually at between 2 and 3 weeks). This suggests that localized virus transmission that we observe is not a specific feature of the RSV A2 isolate, but is likely an inherent property of most RSV strains.

Histological analysis of biopsy material from clinical material has identified RSV-infected cells that have undergone fusion in the lower airway and that resemble syncytia [36]. However, it is not clear if these clinical manifestations are identical to the syncytia that is observed in tissue culture. Irrespective, our analysis indicates that syncytia formation is not a direct consequence of virus transmission in the cell monolayer, but that they arise due to indirect virus-induced effects on the cell physiology that follows advanced infection in the monolayer. The F-protein initiates fusion of the virus envelope and cell membrane during the process of virus entry into non-infected cells. Therefore F protein membrane fusion would be expected to mediate the initial phase of virus transmission that leads to the formation of the virus-infected cell clusters. The absence of syncytia formation during this early phase in virus transmission suggests that although virus-induced membrane fusion may contribute to syncytium formation during the later of infection, it may not be the cause of syncytium formation. Changes in cell physiology that follow extensive virus infection may make a greater contribution towards the gross changes in cell monolayer morphology (i.e. syncytium) that are observed at the advanced stages of virus infection. We have not performed an extensive examination of the signaling networks that are activated in this low moi cell infection model. However in the infected cell clusters we have detected that activation of at least 3 signaling pathways that are implicated in mediating changes in cell physiology. This suggests that these signaling pathways may also play a role in syncytium formation at the advanced stages of virus infection, and their formation is an indirect consequence of cell-to-cell virus transmission. In this context, activation of MAPKp38 and JNK signaling pathways following RSV infection can cause cell monolayer disruption by inducing changes in cell membrane properties [37].

DNA fragmentation occurs at the late stage of apoptosis and necrosis [38] and TUNEL staining in the cell monolayers at 4 and 5 dpi are consistent with RSV-induced cell death at the advanced stages of infection The DNA fragmentation also correlated the increased presence of syncytia formation in the cell monolayer. RSV being able to block apoptosis by PI3K-dependant signaling pathway involving the down-stream effectors AKT and GSK [30], and interestingly RSV virus filament formation and transmission requires activated PI3K [19]. This suggests that cellular changes required for virus particle formation may also prevent cell death during this critical stage of the virus replication cycle. However, TUNEL staining by itself is not able to distinguish between the DNA fragmentation that occurs due to apoptosis or necrosis [38]. Apoptotic cells are characterized by having an intact cell membrane, but the TB-exclusion and LDH assays indicated significant loss of membrane integrity in the RSV-infected cell monolayer starting from 3 dpi. The loss of membrane integrity (from 3 dpi) preceded the DNA fragmentation (at 4 and 5 dpi), and it is not clear if changes in membrane permeability induce cellular changes that eventually leads to cell death. Since DNA fragmentation in a cell with an intact cell membrane is a feature of apoptosis, this suggested that the cell death is due to necrosis rather than by apoptosis.

Purified cell-free RSV particles obtained from permissive cell lines (e.g. HEp2 cells) are usually harvested at the advanced stage of infection when typically there is considerable CPE and cell damage. Furthermore, this process usually requires additional biochemical procedures that facilitate the mechanical release of the budding virus from the infected cells. These cell-released virus particles typically exhibit pleomorphic morphology when they are examined by electron microscopy. Similar pleomorphic RSV particles can also be detected in clinical material (e.g. nasopharyngeal aspirates) following natural infection. On the basis of our low moi cell infection model we can therefore speculate that pleomorphic cell-free virus particles that are present in nasopharyngeal aspirates may also arise due to extensive cell death in tissue following multiple cycles of virus replication. The precise mechanism of RSV transmission from the upper to lower airways of humans has not yet been clearly defined. In the clinical scenario localized virus transmission in the upper airway followed by virus-induced cytotoxicity and the release of virus particles due to the general deterioration of infected cells could provide a mechanism for the dissemination of infectious virus particles; from the initial site of infection to other distant tissues (e.g. located in the lower airway). In addition, RSV transmission between individuals can occur through infected respiratory secretions via fomites [39], and it is reasonable to speculate that the cell-free virus within fomites could provide a mechanism for RSV transmission between infected and non-infected individuals. This implies that blocking the initial localized virus transmission should reduce syncytium formation and the level of cell-free virus, and as a consequence reduce virus dissemination and transmission. This hypothesis will require further examination in primary cell culture systems that more correctly mimic situation in the infected host (e.g. [40]).

Lastly, cell-based model systems are currently an important tool in screening candidate antivirus drugs against RSV. In these assays the efficacy of drug candidate is usually assessed by measuring its capacity to reduce virus infectivity in the tissue culture supernatant of infected cells i.e. to reduce the levels of CF-virus infectivity. However, measuring the plaque size reduction (i.e. reduced virus spread) in addition to the levels of CF-virus infectivity could also facilitate the identification candidate drugs that inhibit RSV transmission.

## Conclusion

In RSV-infected HEp2 cell monolayers we have identified two stages in virus replication. During an early stage in infection there is efficient localized transmission in the monolayer, and the absence of cell-free virus particles. Changes in the F-actin structure within areas of virus infection were observed, which was consistent with reports that F-actin plays a role in mediating virus transmission. However, this was associated with minimal effects on the cell monolayer. From 3 dpi virus-induced changes in the cell monolayer was noted, and these changes correlated with the presence of cell-free virus. Morphological changes in the cell monolayer continued with increased incubation time, leading to syncytial formation, cell-death, and increased levels of cell-free virus. However, even at the later stages of infection the cell-free virus only accounted for less than 1 % of the total virus infectivity. These observations suggest that localized cell-to-cell transmission of RSV is the major route of transmission, but that virus-induced changes in the cell structure occur during advanced virus infection, and it is these structural changes in the cell monolayer that lead to the presence of this low level cell-free virus.

## Methods

### Cells and virus culture

The RSV A2 strain was propagated and prepared as described previously [21]. The HEp2, A549 and MDCK cells were maintained in Dulbecco’s Modified Eagle’s medium (DMEM) (Invitrogen) with 10 % FCS and 1 % penicillin/streptomycin (Invitrogen). MDCK cells were also seeded in Corning Transwell™ inserts according to manufacturer’s instructions. Prior to experimentation the integrity of the cell monolayer was confirmed by measuring the transepithelial electrical resistance (TEER) using an EVOM voltammeter device with STX2 electrodes (WPI, Sarasota, FL, USA). A value of 771 ± 29 Ω/cm2 was routinely obtained from the MDCK cell monolayers prior to infection.

### Low multiplicity of infection

HEp2 cell monolayers that were approximately 95 % confluent were infected with RSV using a multiplicity of infection of 0.0002. Unless otherwise specified this moi was used throughout. Mock-infected and virus-infected cell monolayers were subsequently maintained in DMEM + 2%FCS at 33 °C.

### Isolation of RSV from HEp2 cell monolayers

This was performed basically as described previously [21]. Briefly, HEp2 cell monolayers (90 % Confluence) were infected with RSV using a moi of 0.1 and at 48 hpi the virus was harvested using acid-washed glass beads. All subsequent steps were performed at 4 °C. The virus suspension was clarified by centrifugation (5000 g for 20 min), and the virus was precipitated using 10 % (w/v) PEG 6000 and resuspended in 20 % sucrose in Hanks’ buffered saline solution (HBSS). This was overlaid on a 30 % sucrose cushion (in HBSS) and centrifuged (51,000 g for 1 h). The pellet was then suspended in 20 % sucrose (in HBSS) and overlaid on a discontinuous gradient consisting of 35, 45, and 60 % sucrose prepared in HBSS and centrifuged (1 h at 165,000 g). The opalescent bands at each interface were harvested and analyzed SDS-PAGE and Western Blotting

### Antibodies and specific reagents

The anti-SH, anti-P, anti-M2 anti-M, anti-NS2 and anti-N have described previously [14, 15, 22]. The anti-F was obtained from Joes Melero (Madid, Spain), and the anti-G and anti-RSV were purchased from Abcam and Novacastra respectively. The anti-STAT1, anti-pSTAT1, anti-JNK, anti-pJNK, anti-MAPKp38 and anti-pMAPKp38 were purchased from transduction laboratories. The anti-rabbit and anti-mouse IgG conjugated to Alexa488 and Alexa555 (invitrogen) were used in this study. Stock solution of NSC23766 (Calbiochem) was prepared in distilled water (5 mg/ml) and working concentrations (50 μg/ml) were prepared in DMEM + 2%FCS just prior to use. Y27632 (Calbiochem) was prepared in distilled water (5 mg/ml) and working concentrations (10 μg/ml) were prepared in DMEM + 2%FCS just prior to use.

### Trypan blue staining of cell monolayers

The HEp2 cell monolayers were incubated with trypan blue solution (0.4 % trypan blue in PBS), and after 2 min incubation at 25 °C the cell monolayers were washed with PBS and imaged using an axiovert plate microscope.

### Microplaque titration of RSV infectivity

At each time of infection the recovered viral infectivity was assessed on HEp-2 cells using a microplaque assay essentially as described previously [21] but with minor modifications. At 48 hpi the monolayers were incubated with an anti-RSV (Novacastra) and anti-mouse IgG conjugated to Alexa 488. The stained microplaques were counted using a low-power inverted fluorescence microscope.

### Immunofluorescence microscopy

Cells on 13 mm glass coverslips were fixed and stained with the relevant primary and secondary antibodies as described previously [11]. The stained cells were mounted on slides using Citifluor and visualized using either a Nikon eclipse 80i fluorescence microscope, or a Zeiss Axioplan 2 confocal microscope using appropriate machine settings (Nikon ECLIPSE TE2000-U).

### SDS PAGE and Western blotting

This was performed as described previously [14]. The protein bands were visualized using the ECL protein detection system (Amersham, USA). In all cases the apparent molecular masses were estimated using Kaleidoscope protein standards (Bio Rad, USA).

### Cytotoxicity measurements

Cytotoxicity measurements were performed using the cytotoxicity detection kit, LDH ver 8 (Roche) as recommended by the manufacturer. Values of 0 % and 100 % cytotoxicity were obtained using the recommended control samples. The 0 % value was obtained using tissue culture medium, while the 100 % value was taken using cells treated with 1 % triton-X100 in tissue culture medium. Absorbance values were measured using a Tecan Infinite F200 μ plate reader with i-Control software and using appropriate machine settings and wavelengths.

### TUNEL assay

This was performed using the DeadEnd™ Fluorometric TUNEL system (Promega) following the manufacturer’s instructions. Briefly, cell monolayers on glass coverslips were washed using PBS and fixed with 4 % PFA in PBS for 20 min. The cells were then washed with PBS and permeabilised with 0.2 % triton X 100 in PBS for 5 min. The coverslips were then incubated with the equilibration buffer containing the nucleotide mix and the rTdT Enzyme for 1 h at 37 °C. The reaction was terminated using the stop solution and washed with PBS. The monolayers were then incubated with anti-G and then anti-mouse IgG conjugated to Alexa 488 (1 h incubation for each antibody). The stained cells were washed with PBS and mounted on a glass slide using mounting media and visualized using a Nikon eclipse 80i fluorescence microscope.

## Additional files

Additional file 1: Figure S1. Distribution of the M2-1, P, N, SH, F M and G proteins in the infected cell clusters. (A) HEp2 cell monolayers were infected with RSV using an multiplicity of infection of 0.0002 and at 2 days post-infection (dpi) the cells were fixed and stained using either anti-M2-1, anti-P, anti-N, anti-SH, anti-F, anti-M or anti-G and stained cells were then viewed using fluorescence microscopy (objective x20). **(B)** An infected cells cluster examined at higher magnification (objective x40 magnification) or (objective x100 magnification). The infected cell clusters (long white arrows) are indicated. Inset, an enlarged imaged where virus filaments (short white arrows) are highlighted. (TIF 1152 kb) Additional file 2: Figure S2. Localized virus transmission occurs within RSV-infected MDCK cell monolayers. MDCK cell monolayers were prepared either on **(A)** glass substrate or **(B)** on Transwell^TM^ inserts and infected with RSV using a multiplicity of infection (moi) of 0.0002. At between 1 and 5 days post-infection (dpi) the virus-infected cells were stained using anti-RSV and anti-mouse IgG conjugated to Alexa 488 and viewed using fluorescence microscopy (IF) (objective x20 magnification). The infected cell clusters (open white box), syncytia (black asterisk) and zones of clearing in the cell monolayer (white asterisk) are highlighted. (TIF 2599 kb) Additional file 3: Figure S3. Distribution of the intercellular F-actin and infected clusters at 3 days post-infection (dpi). HEp2 cell monolayers were infected with RSV using a multiplicity of infection of 0.0002 and at 3 dpi the cells were fixed and stained using anti-G and phalloidin-FITC. In **(A)** an infected cell cluster showing phalloidin-FITC staining at the intercellular junctions and **(B)** a cluster of infected cells in which the phalloidin-FITC-stained intercellular junctions between the cells within the cluster are not defined. The increased F-actin staining in the periphery of this cluster is highlighted (white arrows). (TIF 1497 kb) Additional file 4: Figure S4. Assessing the virus infectivity in the fractions harvested from the discontinuous sucrose gradient. The material harvested at each interface (band-1, band-2 and band-3) was diluted in HBSS solution and harvested by ultracentrifugation as described previously [21]. The pelleted material from each fraction was then resuspended in DMEM and used to challenge HEp2 cell monolayers. At 24 h post-infection the HEp2 cells were stained using anti-RSV and examined using fluorescence microscopy (objective x20 magnification). The presence of inclusion bodies are indicated (white arrows). **(i)** Inset shows enlarged image of stained cells in the area highlighted by white box. Anti-RSV mock-infected HEp2 cells are also shown. (TIF 3333 kb) Additional file 5: Figure S5. Trypan blue staining is detected in the HEp2 cell monolayers. HEp2 cell monolayers were either Mock-infected or RSV-infected using an multiplicity of infection of 0.0002 and at between 1 and 5 days post-infection (dpi) the monolayers were stained using trypan blue (0.4 % trypan blue in PBS) for 2 min at 25 °C and imaged using an inverted light microscope (objective x4 magnification). Trypan blue staining in the cell monolayer is highlighted (black star). In plate F extensive cell loss in the HEp2 cell monolayer is also highlighted (white star) at 5 dpi. (TIF 10250 kb)

## Abbreviations

JNK: c-Jun N-terminal kinase
MAPKp38: P38 mitogen-activated protein kinase
RSV: respiratory syncytial virus
STAT: Signal Transducers and Activators of Transcription family of transcription factors
TUNEL: Terminal deoxynucleotidyl transferase dUTP nick end labelling
