# Supplementary figures and images for: Evidence for a biphasic mode of respiratory syncytial virus transmission in permissive HEp2 cell monolayers
Source: Virol J. 2016 Jan 20;13:12. doi: 10.1186/s12985-016-0467-9 (PMC4719537; doi:10.1186/s12985-016-0467-9)

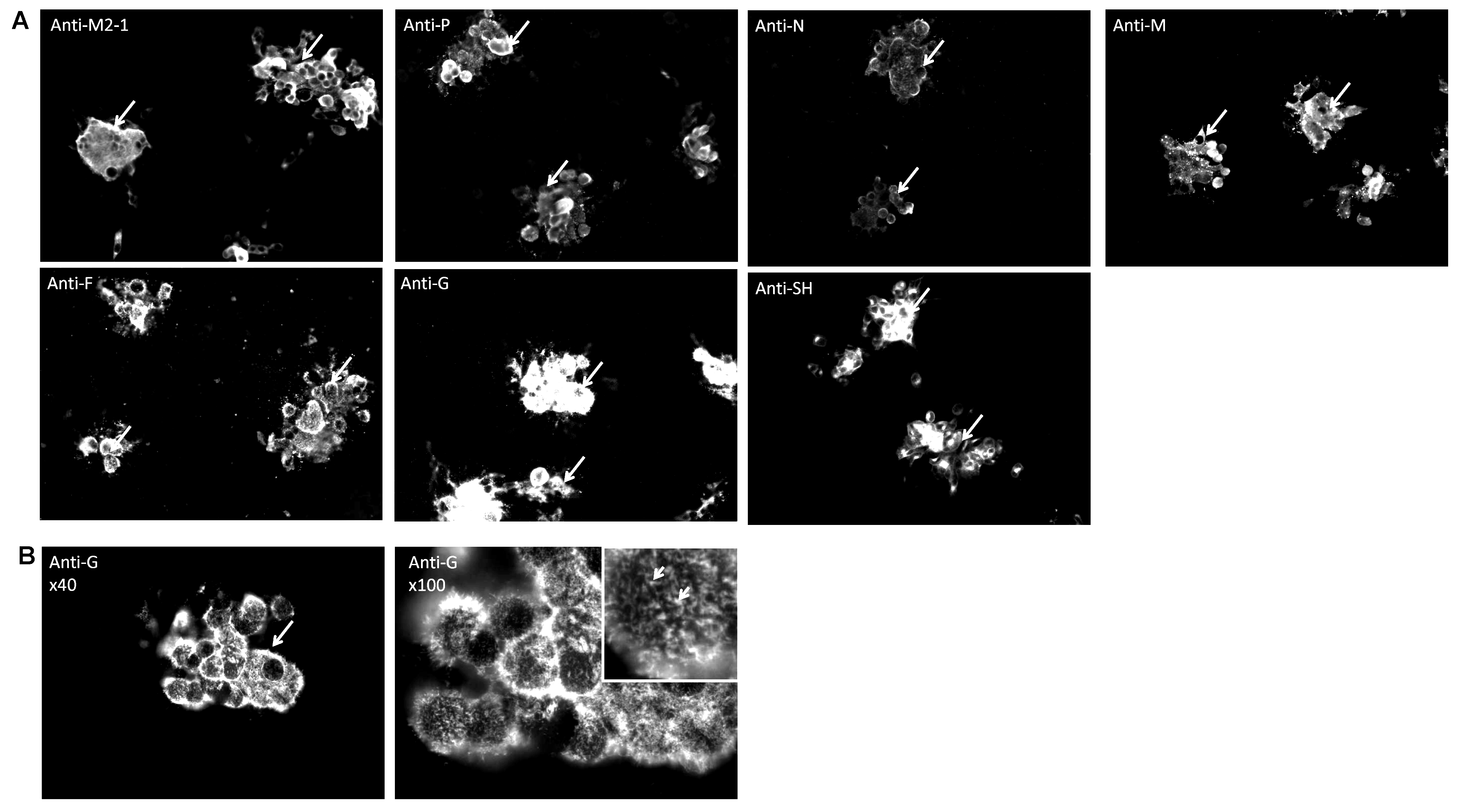

Supplement: Additional file 1: Figure S1. — Distribution of the M2-1, P, N, SH, F M and G proteins in the infected cell clusters. (A) HEp2 cell monolayers were infected with RSV using an multiplicity of infection of 0.0002 and at 2 days post-infection (dpi) the cells were fixed and stained using either anti-M2-1, anti-P, anti-N, anti-SH, anti-F, anti-M or anti-G and stained cells were then viewed using fluorescence microscopy (objective x20). (B) An infected cells cluster examined at higher magnification (objective x40 magnification) or (objective x100 magnification). The infected cell clusters (long white arrows) are indicated. Inset, an enlarged imaged where virus filaments (short white arrows) are highlighted. (TIF 1152 kb) [file 12985_2016_467_MOESM1_ESM.tif]

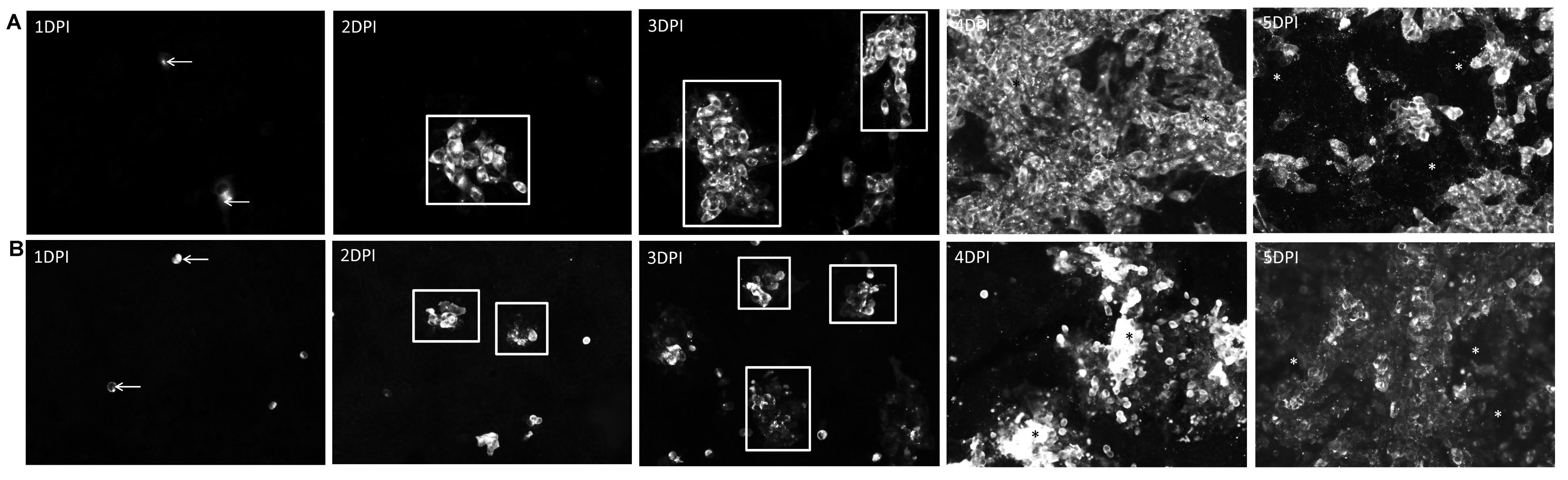

Supplement: Additional file 2: Figure S2. — Localized virus transmission occurs within RSV-infected MDCK cell monolayers. MDCK cell monolayers were prepared either on (A) glass substrate or (B) on TranswellTM inserts and infected with RSV using a multiplicity of infection (moi) of 0.0002. At between 1 and 5 days post-infection (dpi) the virus-infected cells were stained using anti-RSV and anti-mouse IgG conjugated to Alexa 488 and viewed using fluorescence microscopy (IF) (objective x20 magnification). The infected cell clusters (open white box), syncytia (black asterisk) and zones of clearing in the cell monolayer (white asterisk) are highlighted. (TIF 2599 kb) [file 12985_2016_467_MOESM2_ESM.tif]

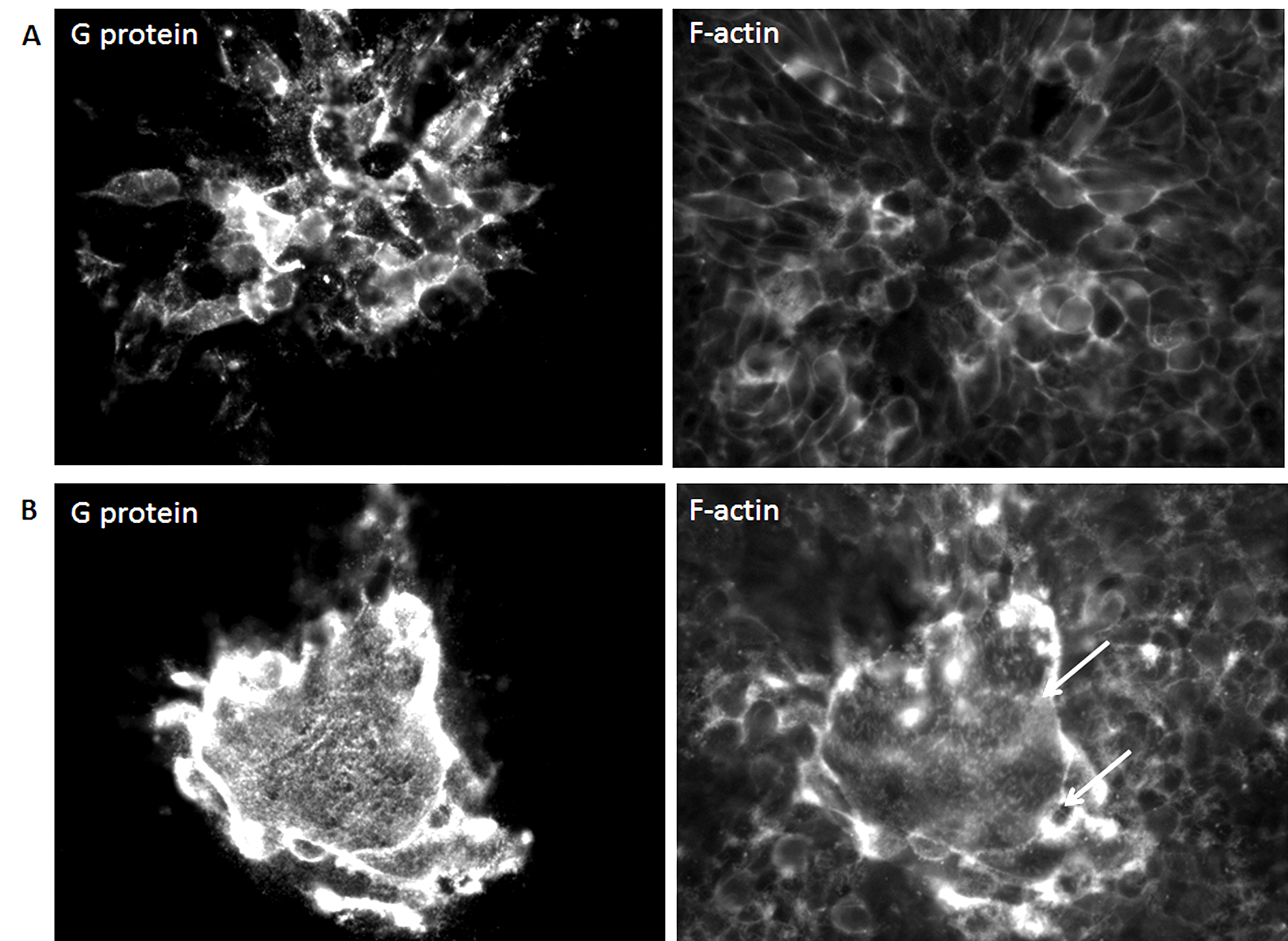

Supplement: Additional file 3: Figure S3. — Distribution of the intercellular F-actin and infected clusters at 3 days post-infection (dpi). HEp2 cell monolayers were infected with RSV using a multiplicity of infection of 0.0002 and at 3 dpi the cells were fixed and stained using anti-G and phalloidin-FITC. In (A) an infected cell cluster showing phalloidin-FITC staining at the intercellular junctions and (B) a cluster of infected cells in which the phalloidin-FITC-stained intercellular junctions between the cells within the cluster are not defined. The increased F-actin staining in the periphery of this cluster is highlighted (white arrows). (TIF 1497 kb) [file 12985_2016_467_MOESM3_ESM.tif]

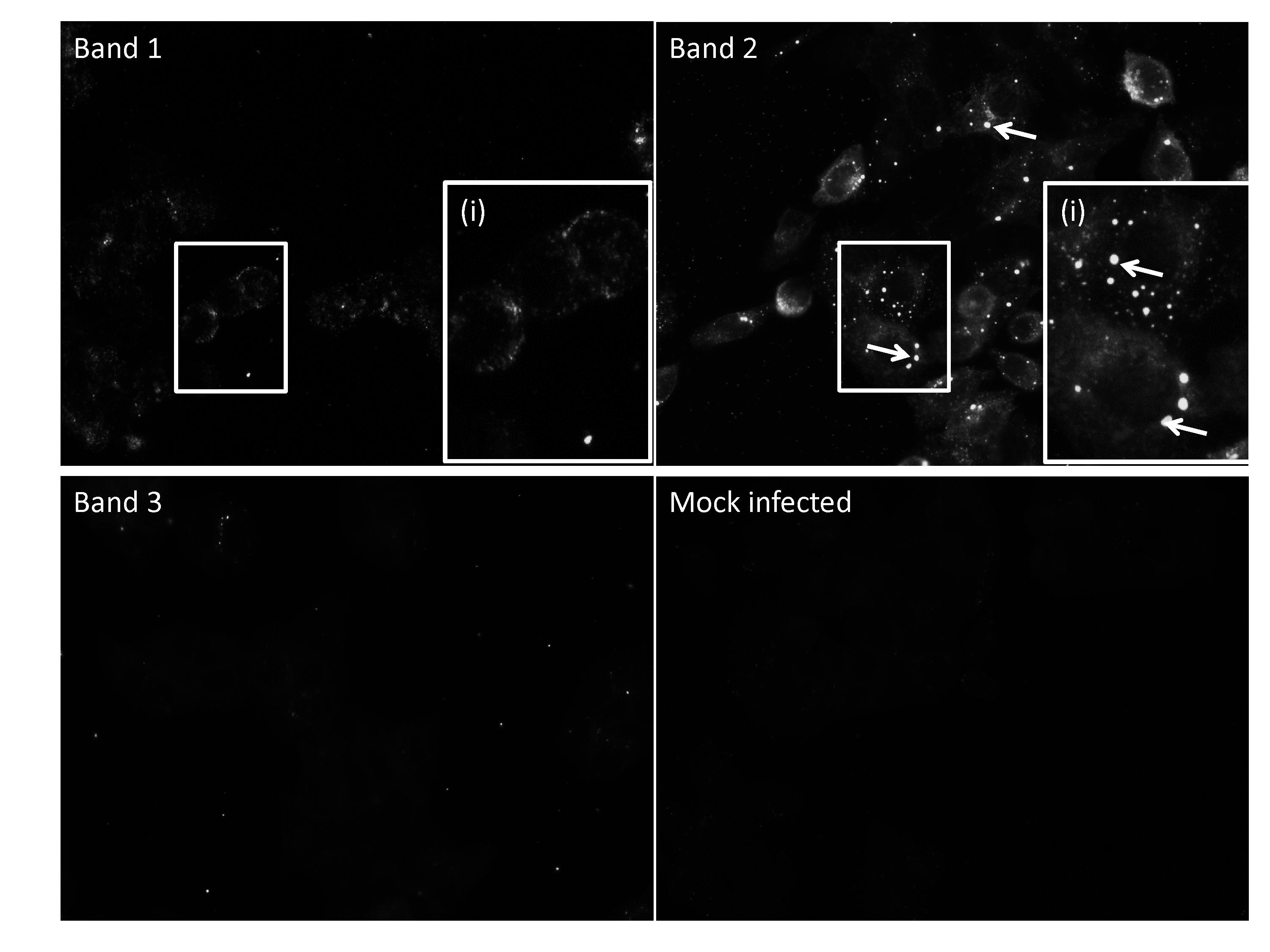

Supplement: Additional file 4: Figure S4. — Assessing the virus infectivity in the fractions harvested from the discontinuous sucrose gradient. The material harvested at each interface (band-1, band-2 and band-3) was diluted in HBSS solution and harvested by ultracentrifugation as described previously [21]. The pelleted material from each fraction was then resuspended in DMEM and used to challenge HEp2 cell monolayers. At 24 h post-infection the HEp2 cells were stained using anti-RSV and examined using fluorescence microscopy (objective x20 magnification). The presence of inclusion bodies are indicated (white arrows). (i) Inset shows enlarged image of stained cells in the area highlighted by white box. Anti-RSV mock-infected HEp2 cells are also shown. (TIF 3333 kb) [file 12985_2016_467_MOESM4_ESM.tif]

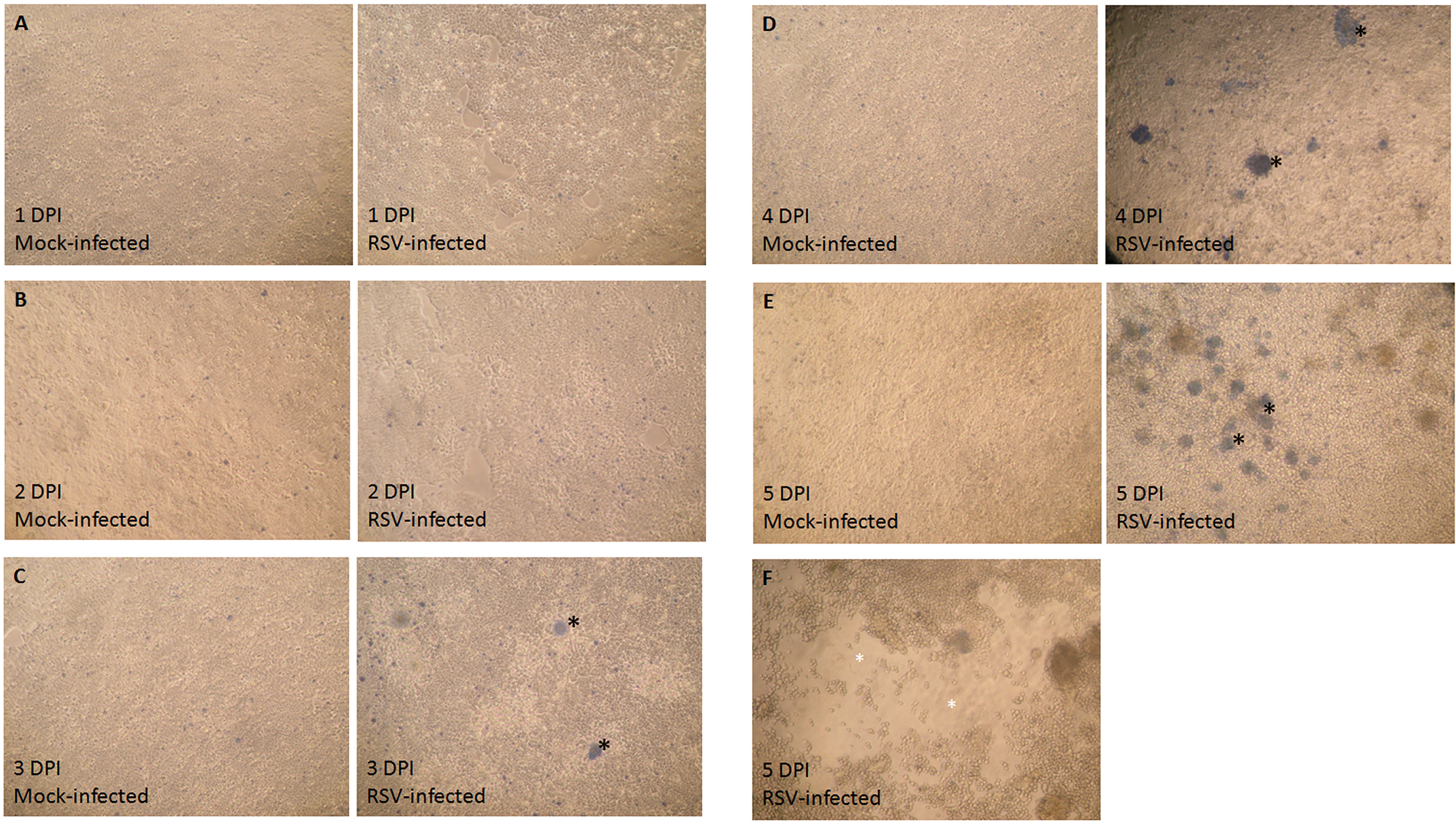

Supplement: Additional file 5: Figure S5. — Trypan blue staining is detected in the HEp2 cell monolayers. HEp2 cell monolayers were either Mock-infected or RSV-infected using an multiplicity of infection of 0.0002 and at between 1 and 5 days post-infection (dpi) the monolayers were stained using trypan blue (0.4 % trypan blue in PBS) for 2 min at 25 °C and imaged using an inverted light microscope (objective x4 magnification). Trypan blue staining in the cell monolayer is highlighted (black star). In plate F extensive cell loss in the HEp2 cell monolayer is also highlighted (white star) at 5 dpi. (TIF 10250 kb) [file 12985_2016_467_MOESM5_ESM.tif]
